# Functional regulations between genetic alteration-driven genes and drug target genes acting as prognostic biomarkers in breast cancer

**DOI:** 10.1038/s41598-022-13835-5

**Published:** 2022-06-23

**Authors:** Li Wang, Lei Yu, Jian Shi, Feng Li, Caiyu Zhang, Haotian Xu, Xiangzhe Yin, Lixia Wang, Shihua Lin, Anastasiia Litvinova, Yanyan Ping, Shangwei Ning, Hongying Zhao

**Affiliations:** 1 College of Bioinformatics Science and Technology, Harbin Medical University, Harbin, 150081 China; 2 Precision Medicine Institute, The First Affiliated Hospital, Sun Yat-Sen University, Guangzhou, China

**Keywords:** Breast cancer, Prognostic markers

## Abstract

Differences in genetic molecular features including mutation, copy number alterations and DNA methylation, can explain interindividual variability in response to anti-cancer drugs in cancer patients. However, identifying genetic alteration-driven genes and characterizing their functional mechanisms in different cancer types are still major challenges for cancer studies. Here, we systematically identified functional regulations between genetic alteration-driven genes and drug target genes and their potential prognostic roles in breast cancer. We identified two mutation and copy number-driven gene pairs (*PARP1-ACSL1* and *PARP1-SRD5A3*), three DNA methylation-driven gene pairs (*PRLR-CDKN1C*, *PRLR*-*PODXL2* and *PRLR*-*SRD5A3*), six gene pairs between mutation-driven genes and drug target genes (*SLC19A1*-*SLC47A2*, *SLC19A1*-*SRD5A3*, *AKR1C3*-*SLC19A1*, *ABCB1*-*SRD5A3*, *NR3C2*-*SRD5A3* and *AKR1C3*-*SRD5A3*), and four copy number-driven gene pairs (*ADIPOR2*-*SRD5A3*, *CASP12-SRD5A3*, *SLC39A11*-*SRD5A3* and *GALNT2*-*SRD5A3*) that all served as prognostic biomarkers of breast cancer. In particular, *RARP1* was found to be upregulated by simultaneous copy number amplification and gene mutation. Copy number deletion and downregulated expression of *ACSL1* and upregulation of *SRD5A3* both were observed in breast cancers. Moreover, copy number deletion of *ACSL1* was associated with increased resistance to PARP inhibitors. *PARP1*-*ACSL1* pair significantly correlated with poor overall survival in breast cancer owing to the suppression of the MAPK, mTOR and NF-kB signaling pathways, which induces apoptosis, autophagy and prevents inflammatory processes. Loss of *SRD5A3* expression was also associated with increased sensitivity to PARP inhibitors. The *PARP1*-*SRD5A3* pair significantly correlated with poor overall survival in breast cancer through regulating androgen receptors to induce cell proliferation. These results demonstrate that genetic alteration-driven gene pairs might serve as potential biomarkers for the prognosis of breast cancer and facilitate the identification of combination therapeutic targets for breast cancers.

## Introduction

The differences in the genetic molecular features of patients, such as DNA methylation, mutation and copy number alterations (CNA), have been shown to be associated with clinical responses to anti-cancer drugs^1–3^. Genomic alterations, including mutations and copy number amplification or deletion, can contribute to genomic instability (GI) in breast cancer (BRCA)^4^. These variations lead to expression dysregulation of oncogenes or suppressor genes, which plays important roles in the growth and survival of breast cancer cells^5^. For example, copy number deletions or mutations of key DNA damage repair genes (such as *BRCA1* and *BRCA2*) and genome caretaker genes (for example, *ATM*, *CHEK2*, and *TP53*) have been found in BRCA patients, and these mutations have been shown to play a crucial role in promoting GI and BRCA tumorigenesis^6–8^. Mutation or over-expression of *EZH2*, a transferase involved in histone methylation, has also been linked to BRCA risk^9,10^, and blocking *EZH2* activity has been shown to slow tumor growth of BRCA^10^. *NBN* gene amplification in ovarian cancer tumors was observed in ovarian cancer patients. *NBN* overexpression in breast and ovarian cancer cells leads to BRCA1-dependent olaparib resistance^11^. Mutation of *BCL2* is associated with resistance to venetoclax in patients with progressive chronic lymphocytic leukemia^12^. In addition to gene mutation, epigenetic changes including DNA methylation aberrations that include hypermethylation and hypomethylation of cancer-related genes can also result in carcinogenesis, and these changes can serve as prognostic biomarkers of various cancers^13,14^. For example, aberrant methylation of the tumor suppressor gene *SFRP1* in BRCA has been directly associated with the loss of *SFRP1* expression and poor prognosis in BRCA^15,16^. *SFRP1* has been demonstrated to antagonize the Wnt signaling pathway and to regulate the transcriptional activity of T-cell factor/lymphocyte enhancer factor, ultimately contributing to tumor initiation and progression^17,18^. DNA methylation-regulated *MCTP1* promotes the drug-resistance of esophageal cancer cells^19,20^. On the other side, drug response gene-associated genomic markers play important roles in serving as guidelines for drug selection of personalized therapies, accurate prognosis and dynamic drug response monitoring^21,22^. For example, epidermal growth factor receptor-2 (*HER2*) is a drug response gene (DRG) for monitoring the humanized monoclonal antibody trastuzumab. Its amplification can promote tumor progression and metastasis, and it is a predictive marker of the treatment benefits from HER2-targeted therapies in breast cancers^23^. Moreover, *PARP1*, a drug response gene of PARP inhibitors, can inhibit BRCA1/2-mediated DNA repair by homologous recombination, thereby inducing tumor cell apoptosis^8,24,25^. The combination of targeting genetic alteration-driven genes and drug response genes represents a novel treatment strategy for cancers. For example, the AT-rich interactive domain 1A gene (*ARID1A*) is mutated in over 50% of ovarian clear cell carcinomas and *ARID1A* mutational status has been shown to correlate with response to an enhancer of zeste homologue 2 (*EZH2*) inhibitor. It has been reported that the inhibition of the EZH2 methyltransferase acts in a synthetic lethal manner in ARID1A-mutated ovarian cancer cells^26^. Therefore, we identified the functional correlation between genetic alteration-driven genes and DRGs in BRCA and characterized their potential prognostic values. In total, we identified 15 genetic alteration-driven gene pairs as prognostic biomarkers of BRCA including 2 both mutation and copy number-driven gene pairs, 3 DNA methylation-driven gene pairs, 6 mutation-driven gene pairs and 4 copy number-driven gene pairs. In particular, copy number amplification and mutation-driven PARP1-associated gene pairs (*PARP1*-*SRD5A3* and *PARP1*-*ACSL1*) significantly correlated with poor overall survival in BRCA through inducing cell proliferation and suppressing the MAPK, mTOR and NF-kB signal pathways. Loss of *SRD5A3* expression was associated with increased sensitivity to a PARP1 inhibitor. Moreover, copy number deletion in *ACSL1* was associated with increased resistance to the PARP1 inhibitor. These results show that genetic alteration-driven gene pairs might serve as potential biomarkers for the prognosis of BRCA and refining combination therapeutic targets for BRCA.

## Results

### Identifying genetic alteration-driven genes in breast cancer

**Figure 1:**
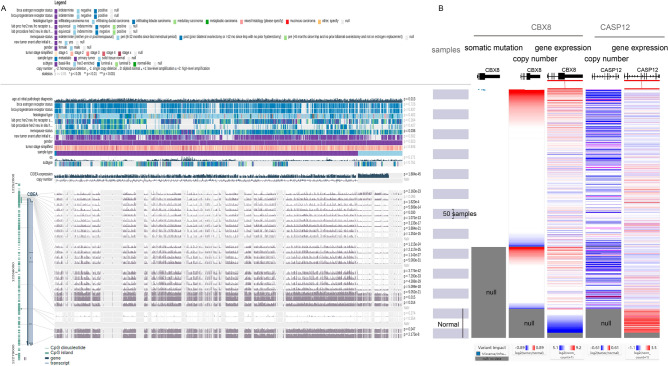
Genetic alteration and gene expression alteration of *CIDEA*, *CBX8* and *CASP12* in BRCA. (**A**) DNA methylation and expression data for the gene *CIDEA* in breast cancer. The DNA methylation data are plotted for each probe separately and the data is linked to the genomic location of the probe. A chi-squared test was used to compare if there was a difference in the methylation distribution between normal and tumor samples. The samples are ordered by the sample type using MEXPRESS. (**B**) Genetic alteration and gene expression alteration of *CBX8* and *CASP12* using UCSC Xena.

Using linear models for microarray data (LIMMA) with a false discovery rate (FDR)-adjusted P value of less than 0.05 and a fold change more than 2 as thresholds, we identified 2388 differentially expressed genes including 1020 upregulated genes and 1368 downregulated genes in BRCA (tumor group = 1085; normal group = 112); 1442 differentially methylated genes, including 670 significantly hypermethylated genes and 770 significantly hypomethylated genes, were also identified using a Student's *t*-test (two-tailed). Among these genes, 101 differentially methylated genes were determined to be DNA methylation-driven genes whose expression showed inverse correlations with their methylation levels (Pearson’s correlation test, adjusted P < 0.05). For example, we found that the gene cell death-inducing DFFA-like effector a (*CIDEA*) exhibited hypermethylation in promoter CpG islands (CGIs) and decreased expression in breast tumor samples relative to normal tissues (LIMMA; fold change = 0.03; FDR = 7.51e−199; Fig. 1A). We also obtained methyl-CpG binding domain protein sequencing (MBD-seq) data on a BRCA cohort consisting of 77 patients and 10 normal controls from Victor Jin et al.^27^ and confirmed the elevated methylation levels of the *CIDEA* gene using Student’s *t*-test (P = 0.0051). CIDEA has been reported to be a member of a class of proapoptotic proteins, and immunotherapeutic treatments have been shown to increase CIDEA expression and apoptosis in BRCA cells^28,29^. Functional analysis showed that DNA methylation-driven genes were significantly enriched for terms including leukocyte transendothelial migration, angiogenesis and negative regulation of tumor necrosis factor production (Fig. S1). We also identified 3348 significantly differentially expressed CNA genes, including 715 copy number amplified genes and 2633 copy number deleted genes, using GISTIC 2.0. Among these genes, 119 CNA-driven genes were found to overlap in the CNA gene list and the differentially expressed gene list. For example, the proapoptotic gene caspase-12 (*CASP12*) was significantly down regulated by simultaneous copy number deletion in BRCA (Fig. 1B). Functional analysis showed that CNA-driven genes were significantly enriched in MAPK signaling pathway, EGFR tyrosine kinase inhibitor resistance and NF-kappa B signaling pathway (Fig. S1). We also identified 8543 significantly mutated genes from The Cancer Genome Atlas (TCGA) by removing silent mutations. Among these, 596 mutation-driven genes were identified via overlapping the somatic mutation gene list with the differentially expressed gene list. Functional analysis showed that mutation-driven genes were significantly enriched for PI3K-Akt signaling pathway, pathways in cancer, MAPK signaling pathway and cell adhesion (Fig. S1). These DNA methylation-driven genes, CNA-driven genes and mutation-driven genes were thus defined as genetic alteration-driven genes in BRCA. There were 8 genes, including angiopoietin-like 5 (*ANGPTL5*), family with sequence similarity 107 member A (*FAM107A*), fibronectin leucine-rich transmembrane protein 2 (*FLRT2*), glycophorin C (*GYPC*), insulin-like growth factor 1 (*IGF1*), junctional adhesion molecule 3 (*JAM3*), sodium channel voltage-gated beta 2 (*SCN2B*) and serpin family B member 9 (*SERPINB9*), which exhibited both DNA methylation alterations and CNA (hypergeometric test P = 0.01). We also identified 46 genes, such as prolactin receptor (PRLR), phosphatidylinositol 3,4,5-trisphosphate-dependent Rac exchanger 1 (*PREX1*), coiled-coil domain containing 8 (*CCDC8*), homeobox A5 (*HOXA5*) and phospholipase C delta 1 (*PLCD1*), which were driven by both DNA methylation alterations and mutation alterations (hypergeometric test P = 9.4e−26). We identified 44 genes (such as *PARP1*, *CBX8*, *EGR3*, *NRG1* and *IL1R1*) that were driven by both CNA and mutation (hypergeometric test P = 2.9e−20). Finally, we identified two genes (*ANGPTL5* and *FAM107A*) that are driven by DNA methylation alteration, CNA and mutation (Fig. S2). For example, *CBX8*, a central part of the polycomb repressive complex 1 (*PRC1*), was significantly upregulated by simultaneous copy number amplification and mutations in BRCA (Fig. 1B). Recent studies have shown that upregulation of *CBX8* can promote BRCA metastasis via the WNK2/MMP2 pathway^30^.

### Identifying functional regulations between genetic alteration-driven genes and drug response genes

**Figure 2:**
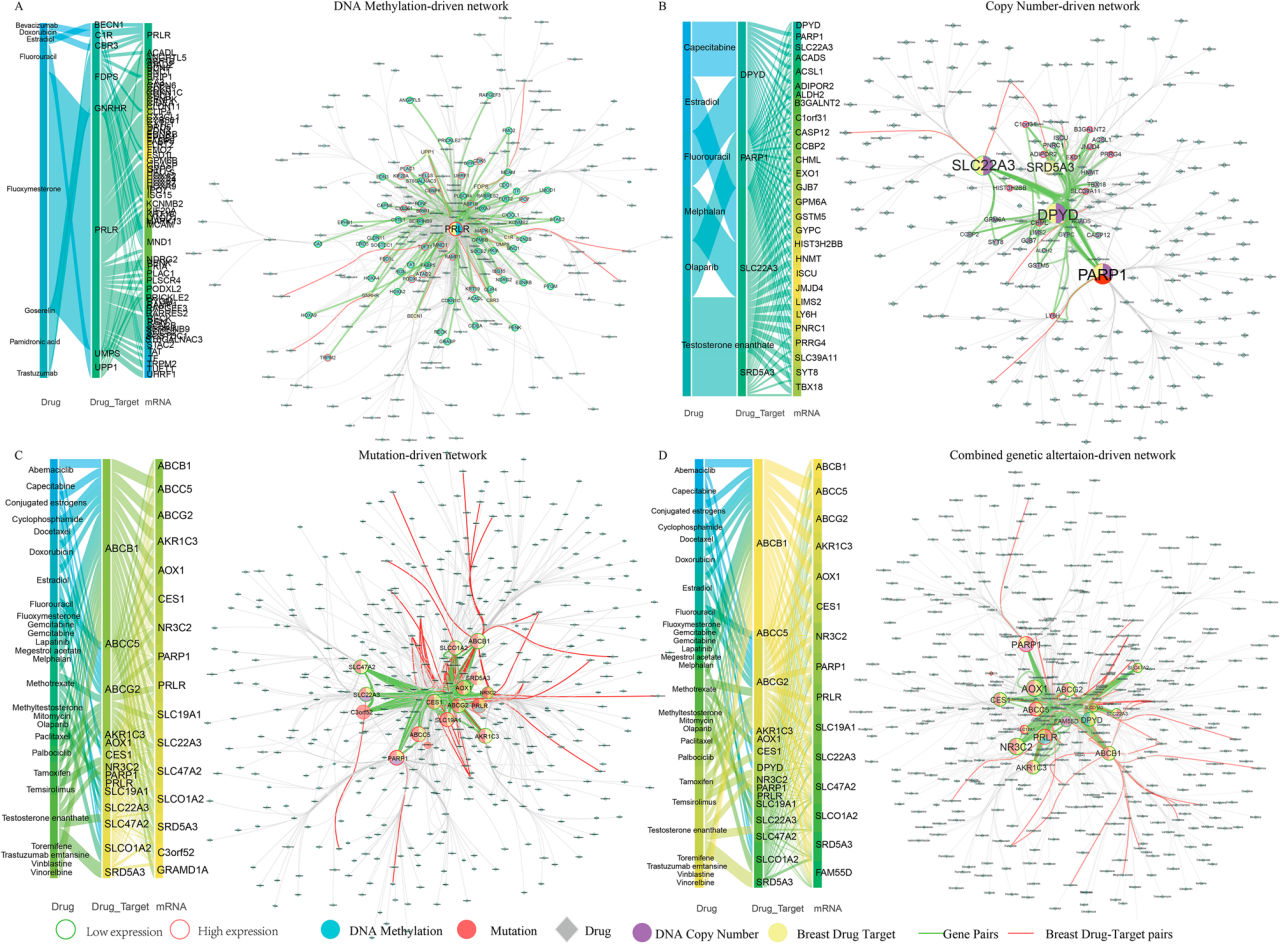
Genetic alteration-driven networks. (**A**) DNA methylation-driven network. (**B**) Copy number-driven network. (**C**) Mutation-driven network. (**D**) Combined genetic alteration-driven network. The node size represents the degree of genes. The green edges represent gene pairs between genetic alteration-driven genes and drug target genes. The red edges represent gene pairs between genetic alteration-driven genes and target genes of breast cancer drugs. The node fill color indicates the genetic alteration and the node edge color corresponds to expression difference.

**Figure 3:**
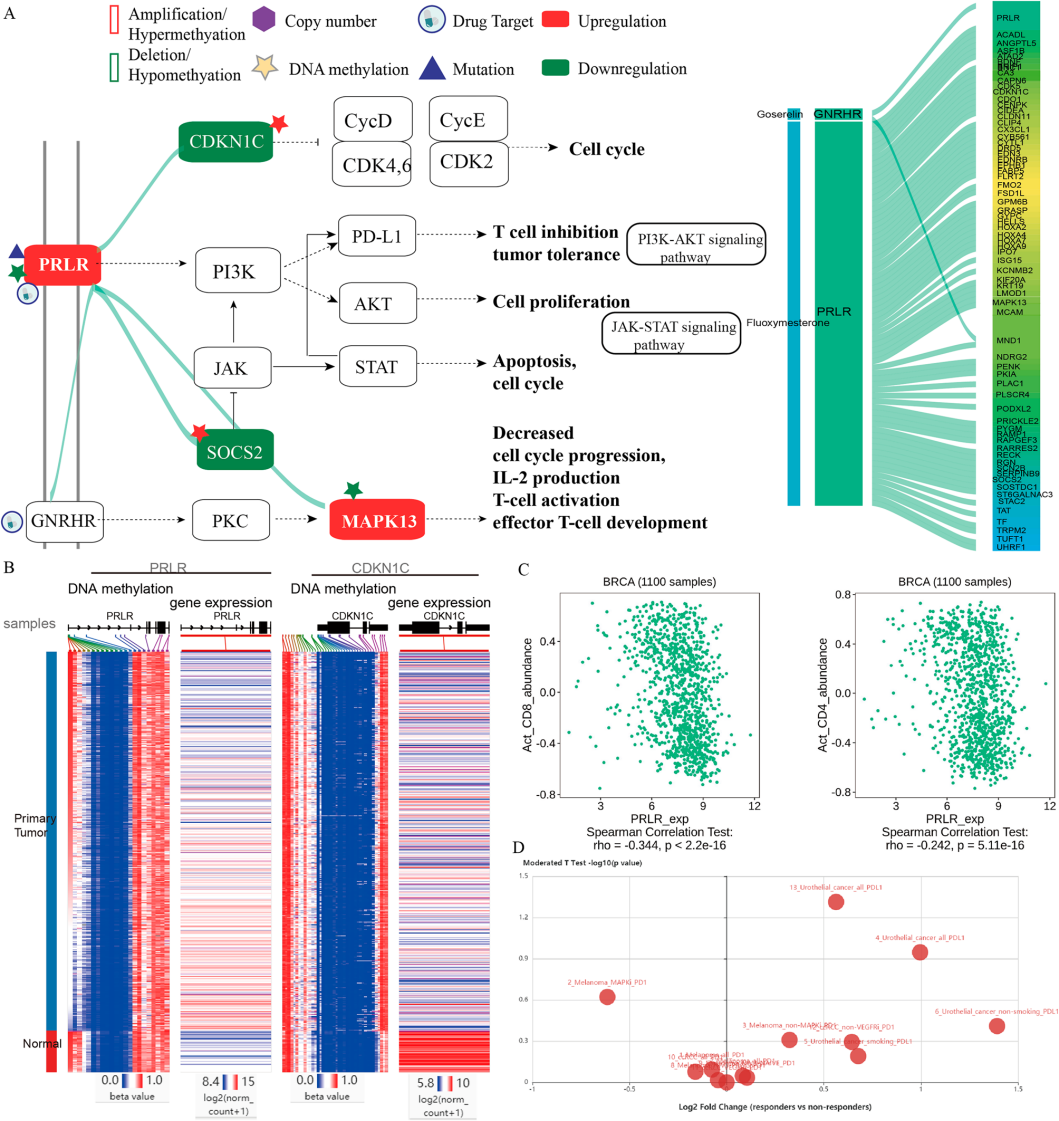
PRLR-associated functional regulations in the methylation alteration-driven network. (**A**) PARP1-associated gene pairs (green line) can regulate cell proliferation, apoptosis, T cell inhibition, and the JAK-STAT and PI3K-AKT signaling pathways^81^. (**B**) Genetic alteration and gene expression alteration of genes using UCSC Xena. (**C**) Spearman’s correlations between of *PRLR* expression and immune cell levels across human breast cancers. (**D**) Expression differences of *PRLR* between responders and non-responders to immunotherapy using TISIDB.

To characterize the functional correlation between genetic alteration-driven genes and DRGs in BRCA, the mutual predictability method was used based on a weighted functional linkage network in visANT (Fig. S3; see “Materials and methods”). The genetic alteration-driven networks including the top 100 functional correlations between genetic alteration-driven genes and DRGs and their associated drugs were used for subsequent analysis (Fig. 2A–D). In the methylation alteration-driven network, we found that genetic alteration-driven gene pairs were related with to eight drugs used in the treatment of BRCA. For example, *PRLR*, a type I cytokine receptor, showed loss of DNA methylation, mutation and upregulated expression in BRCA cells (Fig. 3A,B). Recent studies have shown that blocking PRLR using antibodies such as fluoxymesterone has potent PRLR-specific antitumor activity in cases of BRCA^31^. PRLR-associated gene pairs such as *PRLR-CDKN1C*, *PRLR-SOCS2*, *PRLR-MAPK13* and *PRLR-GNRHR* have been shown to regulate cell proliferation, apoptosis, T cell inhibition, and the JAK-STAT and PI3K-AKT signaling pathways (Fig. 3A). Cyclin dependent kinase inhibitor 1C (*CDKN1C*) showed an increased in DNA methylation and downregulated expression in BRCA (Fig. 3B). This gene is a strong inhibitor of several G1 cyclin/Cdk complexes and a negative regulator of cell proliferation. Suppressor of cytokine signaling 2 (*SOCS2*) showed an increase in DNA methylation and downregulated expression in BRCA, and this cytokine-inducible negative regulator of cytokine receptor signaling has been shown to be negatively associated with activation of the JAK-STAT pathway. Mitogen-activated protein kinase 13 (*MAPK13*), which can inhibit T cell activation, showed a loss of DNA methylation and upregulated expression in BRCA. Additionally, *PRLR* and *SOCS2* could promote the expression of phosphoinositide-3 kinase (PI3K) and programmed death ligand 1 (*PD-L1*), which can protect tumor cells from T cell-mediated immune surveillance, and immune checkpoint blockade (ICB) therapies. *PRLR* has also been shown to have negative associations with the infiltration levels of activated CD8 T cells (Spearman’s correlation test: R = − 0.34, P < 2.2e−16) and activated CD4 T cells (Spearman’s correlation test: R = − 0.24, P = 5.55e−16; Fig. 3C) in BRCA using TISIDB database. Using a cohort of 298 urothelial cancer patient treated with the PD-L1 blockade cancer immunotherapy agent atezolizumab, we found that PRLR showed significant gene expression differences between 68 responders and 230 non-responders (fold change = 1.5, moderated *t*-test P = 0.049; Fig. 3D). This was consistent with a recent study demonstrated that both the tumor antigen *PRLR* and T cell surface CD3 antigen could recruit and activate T cells to kill *PRLR* expressing BRCA cells^31^. In the CNA alteration-driven network, we found that CNA-driven gene pairs were related to six drugs used in the treatment of BRCA, including the pairs *PARP1-ACSL1*, *B3GALNT2-SRD5A3*, *IPOR2-SRD5A3*, *CASP12-SRD5A3* and *PARP1-SRD5A3*. For example, SRD5A3-associated gene pairs such as *ADIPOR2-SRD5A3*, *CASP12-SRD5A3* and *PARP1-SRD5A3* can all regulate cell proliferation and apoptosis. Adiponectin receptor 2 (*ADIPOR2*) showed copy number amplification and upregulated expression in BRCA cells^32^. *ADIPOR2* is a receptor for adiponectin C1Q and collagen domain containing (*ADIPOQ*), an adipocytokine secreted by adipocytes in the breast tumor microenvironment, which negatively regulates cancer cell growth. Recent studies have shown that ADIPOQ treatment can lead to cytotoxic autophagy and tumor growth inhibition in BRCA^33^. The β1,3-N-acetylgalactosaminyltransferase II (*B3GALNT2*) gene showed copy number amplification and upregulated expression in BRCA cells. Studies have also shown that *B3GALNT2* transfers N-acetylgalactosamine (GalNAc) in a β1,3 linkage to N-acetylglucosamine, which plays a critical role in the growth of BRCA cells^34^. In the mutation-driven network, we found that genetic alteration-driven genes pairs were related with 27 drugs used in the treatment of BRCA. For example, SRD5A3-associated gene pairs such as *AKR1C3-SRD5A3*, *PRLR-SRD5A3* and *ABCA1-SRD5A3*, could regulate steroid hormone biosynthesis signaling pathway, invasion, metastasis and acquisition of therapeutic drug resistance, as well as apoptosis. Aldo–keto reductase family 1 member C3 (*AKR1C3*) is known as a hormone activity regulator and prostaglandin F synthase that regulates the occupancy of hormone receptors and cell proliferation^35^. The overexpression of *AKR1C3* has been reported to be correlated with poor prognosis in BRCA^36^. *ABCB1* encodes the multidrug resistance protein (*MDR1*), which is frequently involved in transcriptional fusions in recurrent BRCA. This has important implications for chemotherapy choice in disease relapse and the clinical development of targeted agents^37^. Overexpression of *MDR1* has been associated with olaparib resistance in both cell lines and animal models^38^.

### Functional regulations between genetic alteration-driven genes and drug target genes are associated with disease prognosis in breast cancer

**Figure 4:**
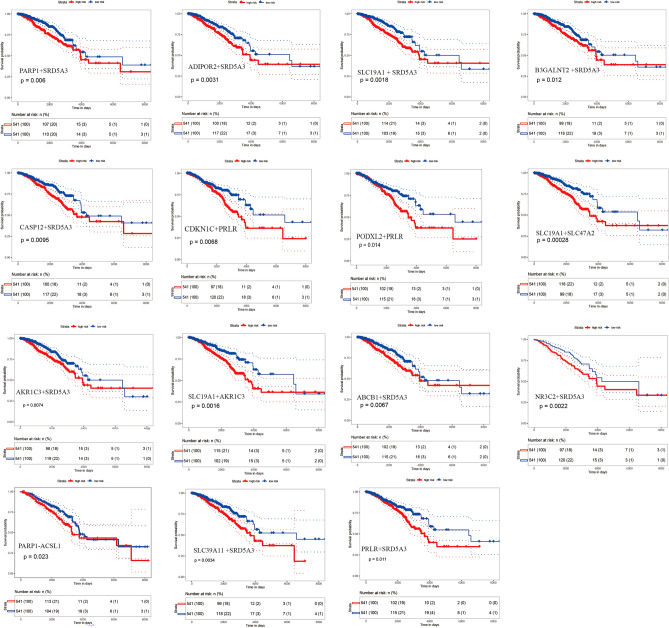
Survival analysis of genetic alteration-driven gene pairs in a TCGA cohort. Comparison of overall survival among patients with high (red) or low (blue) risk scores for each genetic alteration-driven gene pair by Kaplan–Meier analysis (with log-rank values) in a cohort of breast cancer patients from TCGA.

We next sought to evaluate the potentially prognostic value of functional regulations between genetic alteration-driven genes and drug target genes using multivariate Cox regression analysis with the covariates age, estrogen receptor (ER) status, progesterone receptor (PR) status and pathologic stage (see “Materials and methods”). We found 15 gene pairs between genetic alteration-driven genes and drug target genes (*PARP1-ACSL1*, *PARP1-SRD5A3*, *PRLR-CDKN1C*, *PRLR-PODXL2*, *PRLR-SRD5A3*, *ADIPOR2-SRD5A3*, *CASP12-SRD5A3*, *SLC39A11-SRD5A3*, *B3GALNT2-SRD5A3*, *SLC19A1-SLC47A2*, *SLC19A1-SRD5A3*, *AKR1C3-SLC19A1*, *ABCB1-SRD5A3*, *NR3C2-SRD5A3* and *AKR1C3-SRD5A3*), which were able to significantly distinguish patients in high-risk groups from those in low-risk groups in terms of overall survival (Fig. 4, Table 1). This involved 15 genes, including *PARP1*, *ACSL1*, *SRD5A3*, *ADIPOR2*, *SLC39A11*, *B3GALNT2*, *CASP12*, *CDKN1C*, *PRLR*, *PODXL2*, *SLC19A1*, *SLC47A2*, *AKR1C3*, *ABCB1* and *NR3C2*. There were many hub genes in these prognostic gene pairs, such as *PARP1*, *PRLR*, *SRD5A3* and *ABCB1* (Fig. 4). For example, *PRLR* is a hub gene in the DNA methylation-driven network that showed a loss of DNA methylation, mutation and upregulated expression (LIMMA; fold change = 4.19; FDR = 1.56e−82) in BRCA. The higher expression group of PRLR-SRD5A3 [hazard ratio (HR) = 0.66; 95%, confidence interval (CI) 0.47–0.92; log-rank test P = 0.01], *PRLR-CDKN1C* (HR 0.67; 95% CI 0.49–0.94; log-rank test P = 6.8e−03) and *PRLR-PODXL2* (HR 0.70; 95% CI 0.50–0.97; log-rank test P = 0.01) pairs had a significantly decreased overall survival (Fig. 4; Table 1), which increased their prognostic value compared to SRD5A3 (P = 0.03; log-rank test), PRLR (P = 0.19; log-rank test), *CDKN1C* (P = 0.20; log-rank test) or *PODXL2* (P = 0.71; log-rank test) alone (Fig. S4). The multidrug resistance protein 1 (*ABCB1*) is often mutated in BRCA, and is related to the epithelial-to-mesenchymal transition mechanism and cancer metastasis. The higher risk group *ABCB1-SRD5A3* showed significantly shorter survival times than the lower risk group (HR 0.65; 95% CI 0.47–0.91; log-rank test P = 6.7e−03), which increased the prognostic value of this pair compared to *SRD5A3* (P = 0.03; log-rank test) or *ABCB1* (P = 0.11; log-rank test) alone (Fig. S4). These results suggest that genetic alteration-driven gene pairs play important roles in BRCA and can act as potential prognostic biomarkers of BRCA.

**Table 1:** The information of pairs of genetic alteration-driven genes and drug response genes associated with survival in breast cancer.

Gene pairs	Genetic alterations	Univariate cox analysis				Multivariate cox analysis			
		P.value ^a^		HR (95%CI)		P.value ^b^		FDR	HR (95%CI)
**PARP1–ACSL1**									
Low or High	PARP1: Mutation; copy number amplification; High expression ACSL1: copy number deletion; Low expression	0.024		0.72(0.52–1.004)		0.021		0.042	0.68(0.49–0.94)
**PARP1–SRD5A3**									
Low or High	PARP1: Mutation; copy number amplification; High expression SRD5A3: High expression	0.0064		0.63(0.46–0.8794)		0.0046		0.028	0.62(0.45–0.86)
**ADIPOR2–SRD5A3**									
Low or High	ADIPOR2: copy number amplification; High expression SRD5A3: High expression	0.0034		0.61(0.442–0.8508)		0.0052		0.028	0.63(0.45–0.87)
**SLC39A11–SRD5A3**									
Low or High	SLC39A11: copy number amplification; High expression SRD5A3: High expression	0.0037		0.62(0.4434–0.8544)		0.0092		0.029	0.65(0.47–0.90)
**B3GALNT2–SRD5A3**									
Low or high	B3GALNT2: copy number amplification; High expression SRD5A3: High expression	0.013		0.66(0.4784–0.9172)		0.011		0.029	0.65(0.47–0.91)
**CASP12–SRD5A3**									
Low or High	CASP12: copy number Deletion; Low expression SRD5A3: High expression	0.01		0.65(0.4695–0.9024)		0.018		0.040	0.67(0.48–0.93)
**CDKN1C**–**PRLR**									
Low or High	CDKN1C: DNA Hypermethyation; Low expression PRLR: DNA Hypomethyation; Mutation; High expression	0.0073		0.64(0.4611–0.8864)		0.019		0.031	0.67(0.49–0.94)
**PODXL2**–**PRLR**									
Low or High	PODXL2: DNA Hypomethyation; High expression PRLR: DNA Hypomethyation; Mutation; High expression	0.015		0.67(0.4790.9232)		0.031		0.031	0.70(0.50–0.97)
**SLC19A1**–**SLC47A2**									
Low or High	SLC19A1: Mutation; High expression SLC47A2: Mutation; Low expression	0.00035		0.54(0.3849–0.7566)		0.0016		0.032	0.58(0.41–0.81)
**SLC19A1**–**SRD5A3**									
Low or High	SLC19A1: Mutation; High expression SRD5A3: High expression	0.0021		0.59(0.4239–0.8263)		0.005		0.033	0.62(0.44–0.86)
**AKR1C3**–**SLC19A1**									
Low or high	AKR1C3: Mutation; Low expression SLC19A1: Mutation; High expression	0.0018		0.59(0.4186–0.8199)		0.0067		0.034	0.63(0.45–0.88)
**ABCB1**–**SRD5A3**									
Low or High	ABCB1:Mutation; Low expression SRD5A3: High expression	0.0072		0.64(0.4615–0.8862)		0.011		0.038	0.65(0.47–0.91)
**PRLR**–**SRD5A3**									
Low or High	PRLR: DNA Hypomethyation; Mutation; High expression SRD5A3: High expression	0.012		0.66(0.4717–0.91)		0.012		0.038	0.66(0.47–0.92)
**NR3C2**–**SRD5A3**									
Low or high	NR3C2: Mutation; Low expression SRD5A3: High expression	0.0024		0.60(0.4335–0.8355)		0.013		0.038	0.66(0.47–0.92)
**AKR1C3**–**SRD5A3**									
Low or high	AKR1C3: Mutation; Low expression SRD5A3: High expression	0.0079		0.64(0.46–0.89)		0.016		0.041	0.67(0.48–0.93)

HR: hazard ratio.

^a^P.value: Univariate Cox regression analysis.

^b^P.value: Multivariate Cox regression analysis (including age, ER, PR, pathological stage), FDR: FDR correction for P.value^b^.

### Genetic alteration-driven PARP1-associated functional regulations could be potential targets for combination therapy and prognostic markers in BRCA

**Figure 5:**
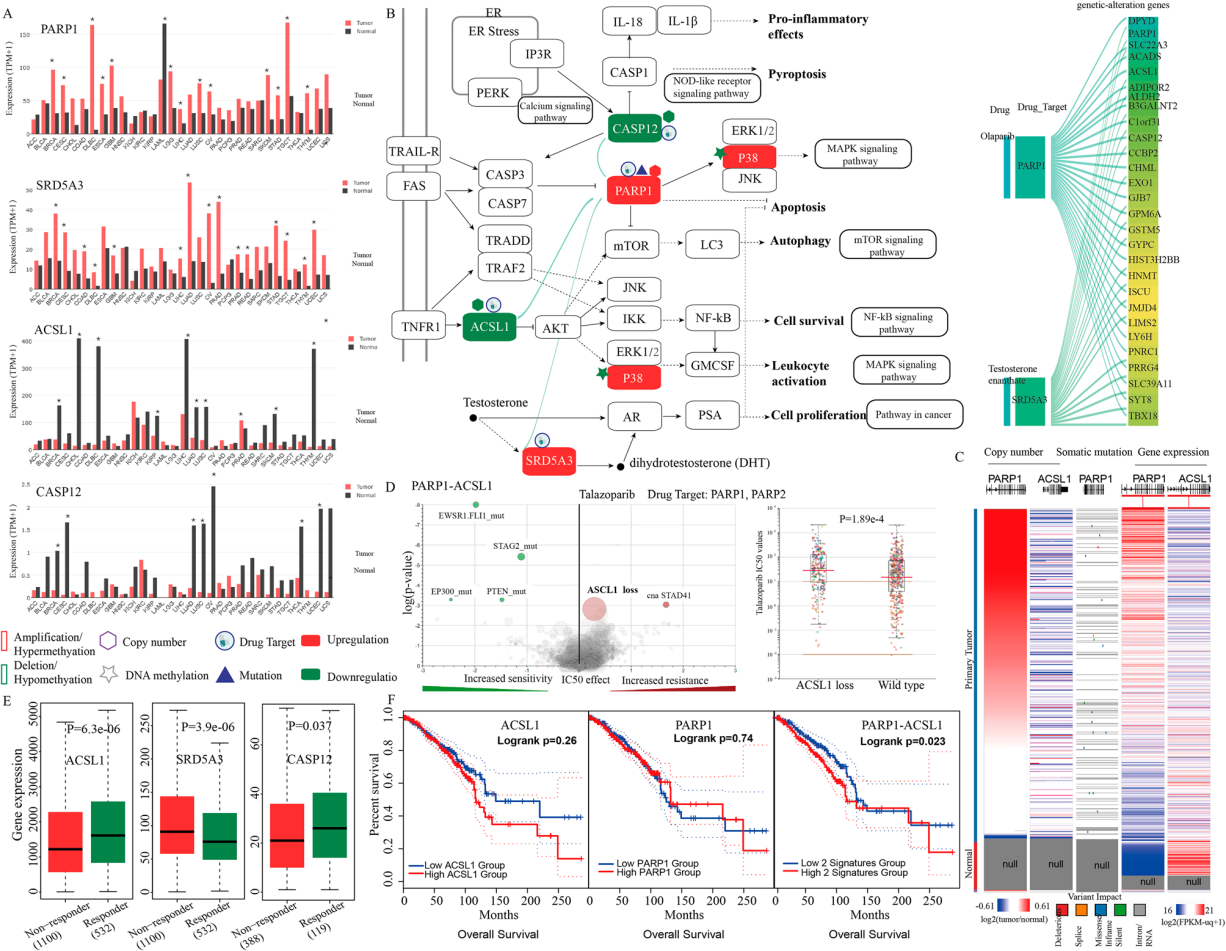
PARP1-associated functional regulations could be potential targets for combination therapy and prognostic markers of breast cancer. (**A**) The gene expression profile across all tumor samples and paired normal tissues. Each dot represents the expression of each sample. Asterisks represent FDR < 0.05. (**B**) PARP1-associated functional regulations (green line) affect multiple important biological pathways^81^. (**C**) Genetic alteration and gene expression alteration of genes using UCSC Xena. (**D**) Association between genetic alteration of genes and drug sensitivity of PARP1 inhibitor analyzed using ANOVA based on GDSC. The size of each point is proportional to the number of altered cell lines (*ASCL1* loss, P = 1.49e−3). The magnitude of the effect that each genetic event has on cell line IC_50_ values in response to the drug. The effect size is proportional to the difference in the mean IC_50_ between wild-type and altered cell lines. Numbers less than 0 indicate drug sensitivity, and numbers greater than 0 indicate drug resistance. (**E**) Expression levels of *ACSL1*, *CASP12* and *SRD5A3* in breast cancer patients in relation to response to chemotherapy treatment. Patients were classified as responder or nonresponder according to their 5-year relapse-free survival. P values were calculated using Mann–Whitney U test. (**F**) Kaplan–Meier curve based on the expression status of one gene or a multi-gene signature in BRCA.

Differences in molecular features including mutation, CNA and DNA methylation can explain the interindividual variability in response to anti-cancer drugs between cancer patients. We therefore sought to investigate the association between genetic alteration-driven gene pairs and treatment efficacy and prognostic indicators. For example, we identified 28 genetic alteration-driven PARP1-associated functional regulations (such as *PARP1-ACSL1*, *PARP1-SRD5A3* and *PARP1-CASP12*) in BRCA. *RARP1* (poly(ADP-ribose)-polymerase 1) is upregulated by simultaneous copy number amplification and gene mutation of *RARP1* in BRCA (Fig. 5A–C). *RARP1* can regulate apoptosis, autophagy, and the MAPK and mTOR signaling pathways through P38, ERK1/2, JNK, mTOR and LC3 (Fig. 5B). Recent studies have shown that PARP1 can promote cancer progression through impacting on DNA repair, apoptosis inhibition, and maintaining the activity of the MAPK signaling pathway^39^. Clinically speaking, PARP1 inhibitors, such as olaparib, have been approved by the FDA or are currently in clinical trials for the treatment of advanced breast and ovarian cancers^40^. The PARP1-associated gene *ACSL1* showed copy number deletion and downregulated expression in BRCA (Fig. 5A–C). *ACSL1* regulates AKT, P38, ERK1/2, JNK, NF-κB and GMCSF to activate inflammatory processes, MAPK signaling and the NF-κB signaling pathway (Fig. 5B). Recent studies have shown that ACSL1 can significantly affect phosphorylation of P38, ERK1/2, and NF-κB in BRCA MDA-MB-231 cells. *ACSL1* has also been reported to play a crucial role in the regulation of *GMCSF* production, which is associated with the inflammatory process that is involved in tumor growth ^41^. Simultaneous suppression of the MAPK, NF-κB and mTOR signaling pathways have indeed been shown to inhibit migration, invasion and have anti-inflammatory effects in multiple cancer types^42–44^. To identify the association between *ACSL1* and the drug response of the PARP1 inhibitor talazoparib, we perform an analysis of variance (ANOVA) using data from the Genomics of Drug Sensitivity Cancer (GDSC) database to correlate drug response [inhibitory concentration (IC_50_) values of talazoparib] with genetic alterations (mutations and CNA) in cancer cells. To this end, we found that copy number deletion in *ACSL1* was associated with increased resistance to PARPi talazoparib (two-way ANOVA P = 1.49e−3, Fig. 5D). The talazoparib IC_50_ value was markedly increased in pan-cancer samples that had ASCL1 copy number deletion (two-way ANOVA P = 1.89e−4, Fig. 5D). Moreover, based on expression data from 1632 BRCA patients treated with chemotherapy and using the response data from Balázs Győrffy et al.^45^, we found that patients who did not respond to therapy by means of relapse-free survival at 5 years showed lower expression of ACSL1 compared with responders (moderated *t*-test P = 6.3e−6; Fig. 5E). We also found that patients with higher expression of the functional regulation unit *PARP1-ACSL1* significantly correlated with poorer overall survival (multivariate cox analysis FDR = 0.04; HR 0.68; 95% CI 0.49–0.94; log-rank test P = 0.023; Fig. 5F). However, there was no prognostic significance of the individual genes *PARP1* (P = 0.74; log-rank test) or *ACSL1* (P = 0.26; log-rank test). These results indicated that combination targeting of *PARP1* and *ACSL1* may be a potential therapeutic strategy to improve the overall survival in BRCA through suppressing the MAPK, mTOR and NF-kB signal pathways to induce apoptosis, autophagy and prevent the inflammatory processes involved in cancer metastasis. Indeed, a recent study showed that the combination of PARP inhibitors (such as olaparib) and metformin could increase the efficacy of PARP inhibitors and tumor sensitivity to immunotherapy in triple-negative BRCA^46^. Metformin has been reported to increase the expression of *ACSL1* mRNA to relieve cardiomyocyte injury based on data from the comparative toxicogenomics database (CTD)^47^. Additionally, PARP1-associated gene SRD5A3 (steroid 5 alpha-reductase 3) was found to be significantly up-regulated (LIMMA; fold change = 2.57; FDR = 7.73e−127) in human BRCA. *SRD5A3* can convert testosterone to dihydrotestosterone and suppress apoptosis and induces cell proliferation through regulating androgen receptor (AR) and kallikrein-related peptidase 3 (*KLK3*) or *PSA* (Fig. 5B). Indeed, knockdown of *SRD5A3* expression could inhibit the growth and cell proliferation of prostate cancer and hepatocellular carcinoma cells^48,49^. To characterize whether the PARP1-associated gene *SRD5A3* could modulate sensitivity to PARP inhibitor (PARPi) treatment, we obtained data from a genome-wide clustered regularly interspaced short palindromic repeats (CRISPR) knockout screen with PARP1 inhibitor olaparib in HeLa cells (GSE145743) ^50^. Using DrugZ, which is an algorithm to identify synergistic and inhibitory chemical interactions from CRISPR screens^51^, we found that loss of *SRD5A3* expression was associated with increased sensitivity to the PARPi olaparib (P = 0.03), indicating a synthetic lethal interaction between *PARP1* and *SRD5A3* with PARPi treatment. Combination inhibition of *PARP1* and *SRD5A3* might therefore be a potential therapeutic strategy for BRCA that acts through suppressing cell proliferation and inducing apoptosis. Accordingly, patients with higher expression of *PARP1-SRD5A3* were significantly correlated with poorer overall survival in BRCA (log-rank test P = 0.006; multivariate cox P = 0.005, Fig. 4). The *PARP1-SRD5A3* pair (multivariate cox analysis FDR = 0.03; HR 0.62; 95% CI 0.45–0.86; log-rank test P = 0.006) increased the prognostic value compared to *SRD5A3* (P = 0.03; log-rank test) or *PARP1* (P = 0.74; log-rank test) alone (Fig. S3). Therefore, the genetic alteration-driven gene pairs *PARP1-ACSL1* and *PARP1-SRD5A3* might serve as potential biomarkers for the prognosis and combination therapy of BRCA.

In addition, *CASP12*, a proapoptotic gene, is an upstream negative regulator of *PARP1*. *CASP12* was significantly downregulated by simultaneous copy number deletion in BRCA (fold change = 2.57, FDR = 7.73e−127; Fig. 5A). *CASP12* plays important roles in both downregulating inflammation through repressing the proinflammatory cytokines IL-18 and IL-1β and executing ER stress-triggered apoptosis through regulating *CASP3*, *CASP7* and *PARP1* (Fig. 5B). Both of *CASP12* and *PARP1* are targets of thymoquinone and paclitaxel based on the CTD database. The combination of thymoquinone and paclitaxel has also been reported to significantly induce apoptosis and inhibit tumor growth compared to each agent alone by affecting the expression and the activity of Caspase-3, Caspase-7, and Caspase-12 in BRCA cells^52^. The PARP1 inhibitor lapatinib plus paclitaxel reduced the risk of progression compared with paclitaxel alone (HR 0.44; P < 0.0001)^53^. Using expression data from 1632 BRCA patients with chemotherapy and response data from Balázs Győrffy et al.^45^, we found that patients who did not respond to therapy showed higher expression levels of *SRD5A3* (moderated *t*-test P = 3.9e−6), but lower expression levels of *CASP12* compared with responders (moderated *t*-test P = 3.7e−2; Fig. 5E). These results establish functional links between genetic alteration-driven genes *PARP1*, *ACSL1*, *CASP12* and *SRD5A3*, could be potential targets for combination therapy and prognostic markers for BRCA that function by partly inducing apoptosis and anti-inflammatory effects in BRCA.

## Discussion

Here, we identified genetic alteration-driven genes for BRCA, including 101 methylation alteration-driven genes, 119 CNA-driven genes and 596 mutation-driven genes. Then, we systematically identified genetic alteration-driven gene pairs and constructed genetic alteration-driven networks including genetic alteration-driven genes, DRGs and their associated drugs for BRCA. There were many hub genes in these genetic alteration-driven networks, including *PARP1*, *PRLR*, *SRD5A3* and *ABCB1*, which are important for BRCA. In the methylation alteration-driven network, *PRLR* showed loss of DNA methylation, mutation and upregulated expression in BRCA cells. PRLR-associated gene pairs such as *PRLR*-*CDKN1C*, *PRLR*-*SOCS2*, *PRLR*-*MAPK13* and *PRLR*-*GNRHR* could regulate cell proliferation, apoptosis, T cell inhibition, and the JAK-STAT and PI3K-AKT signaling pathways. The *PRLR-SOCS2* pair could promote the expression of PI3K and PD-L1, which could protect tumor cells from T cell-mediated immune surveillance, and ICB therapies. In the CNV alteration-driven network, SRD5A3-associated gene pairs such as *ADIPOR2*-*SRD5A3*, *CASP12*-*SRD5A3* and *PARP1*-*SRD5A3* could regulate cell proliferation and apoptosis. In the mutation-driven network, gene pairs such as *AKR1C3*-*SRD5A3*, *PRLR*-*SRD5A3* and *ABCA1*-*SRD5A3* could regulate the steroid hormone biosynthesis signaling pathway, invasion, metastasis, the acquisition of therapeutic drug resistance and apoptosis.

We identified 15 genetic alteration-driven gene pairs as independent prognostic biomarkers of BRCA, which included two mutation and copy number-driven gene pairs (*PARP1*-*ACSL1* and *PARP1*-*SRD5A3*), three DNA methylation-driven gene pairs (*PRLR*-*CDKN1C*, *PRLR*-*PODXL2* and *PRLR*-*SRD5A3*), six gene pairs between mutation-driven genes and drug target genes (*SLC19A1*-*SLC47A2*, *SLC19A1*-*SRD5A3*, *AKR1C3*-*SLC19A1*, *ABCB1*-*SRD5A3*, *NR3C2*-*SRD5A3* and *AKR1C3*-*SRD5A3*), and four copy number-driven gene pairs (*ADIPOR2*-*SRD5A3*, *CASP12*-*SRD5A3*, *SLC39A11*-*SRD5A3* and *GALNT2*-*SRD5A3*). For example, *PRLR* was a hub gene in the DNA methylation-driven network that showed a loss of DNA methylation, mutation and upregulated expression in BRCA. The *PRLR* has been reported to control cell proliferation, migration, and inhibit apoptosis^54–59^. Disruption of *PRLR* signaling pathways have also been linked to tumorigenesis and BRCA development^60^. In accordance, suppression of PRLR expression by shRNA reduced the growth, invasiveness and tumourigenicity of breast cancer T47D cells^61^, which highlights *PRLR* as a therapeutic target for breast cancer^62,63^. Targeting both the tumor antigen *PRLR* and the T cell surface CD3 antigen could recruit and activate T cells to kill *PRLR* expressing breast cancer cells^31^. *SOCS2* showed a gain of DNA methylation and downregulated expression in breast cancers. Studies have showed that *SOCS2* is JAK-STAT regulator and plays a critical regulatory role in antitumor immunity by priming T cells^64,65^. *CDKN1C* showed gain of DNA methylation and downregulated expression in breast cancers. Previous evidence has shown that downregulated *CDKN1C* correlates with poor overall survival, immune infiltration and therapeutic response in breast cancer patients^66,67^. The higher expression group of *PRLR*-*SRD5A3*, *PRLR*-*CDKN1C* and *PRLR*-*PODXL2* pairs had significantly shorter survival times than lower expressers. In particular, *RARP1* was found to be upregulated by simultaneous copy number amplification and gene mutation, which regulates apoptosis, autophagy, and the MAPK and mTOR signaling pathways, leading to mammary microcalcification. It has been demonstrated that mammary microcalcification is not only the earliest detectable radiological sign for BRCA screening but the phenomenon, ectopic breast mineralization, may reflect the underling events during mammary carcinogenesis. The prognostic relevance of genetic alteration-driven gene pair signatures, together with their mRNA quantitation, might result from relevant biological processes that contribute to the molecular heterogeneity of human BRCA^35^. Therefore, breast microcalcification is plausible as having prognostic significance in BRCA patients and is associated with unfavorable genetic and molecular characteristics, complex subgross morphology, high histology tumor grade, increased metastatic potential, decreased disease-free and overall survival and hence the choice of treatment plan^68,69^. *RARP1* is upregulated by simultaneous copy number amplification and gene mutation of the *RARP1* in breast cancers. *PARP1* has been reported to be overexpressed and to be associated with poor overall survival of breast cancer patients ^70,71^. Recent findings have showed that the activation of *PARP1* increases the transcription of some proliferation and DNA repair genes in breast cancer cells, which enables cancer cells to rapidly divide and resist DNA damaging agents^72^. In addition, inhibition of PARP1 can cause a significant reduction in tumor growth as shown by a xenograft experiment in MCF-7 cells^73^, and this can act as a novel and promising radiosensitization strategy in inflammatory breast cancer^74^. PARP1 inhibitors have been approved by the FDA or are in clinical trials for the treatment of advanced BRCA. We found that PARP1-associated gene pairs were associated with sensitivity to PARPi and significantly correlated with overall survival in BRCA. For example, copy number deletion in *ACSL1* was associated with increased resistance to PARP1 inhibitors. *ACSL1* has been reported to play a crucial role in the regulation inflammatory process that is involved in tumor growth and metastasis in BRCA by regulating lipid metabolism and patients. High *ACSL1* expression has also been reported to be correlated with poor prognosis in BRCA^75^. Loss of *SRD5A3* expression was associated with increased sensitivity to PARP inhibitors. Representative immunohistochemistry images confirmed that *SRD5A3* protein expression was high in BRCA tissues and this high *SRD5A3* expression was related to poorer prognosis^76^. The *PARP1*-*ACSL1* and *PARP1*-*SRD5A3* pairs significantly correlated with poor overall survival in BRCA through inducing apoptosis, preventing the inflammatory processes and inducing cell proliferation, respectively. These results establish functional links between the genetic alteration-driven genes *PARP1*, *ACSL1* and *SRD5A3*, which could be potential targets for combination therapy and prognostic markers in BRCA by partly inducing apoptosis and anti-inflammatory effects in BRCA.

## Materials and methods

### Identification of genetic alteration-driven genes in breast cancer

We used RNA-sequencing (RNA-seq) data from 1085 patients and 112 normal samples from TCGA. We used LIMMA (a Bioconductor package in R), which uses a linear model to estimate the mean and variance of gene expression in different groups, to perform differential analysis. We thus identified differentially expressed genes between BRCA and normal samples with the threshold for FDR-adjusted P value < 0.05 and a fold change > 2. Genome-wide DNA methylation data on BRCA were measured using the Infinium HumanMethylation27 BeadChip. The differences in DNA methylation levels (β value) of gene promoters between BRCA and normal samples were analyzed using a Student's *t*-test. Genes showing differentially methylated regions with an FDR < 0.05 and inverse correlations between expression and methylation were termed DNA methylation-driven genes. CNA were assessed using SNP 6.0 Arrays and level 3 copy number data from the TCGA data portal^77^. Identification of significant copy number changes in BRCA were based on GISTIC 2.0, which is software designed for discovering new cancer genes targeted by somatic copy number alterations (SCNA)^77^. GISTIC 2.0 was used by setting the confidence level to 99% for a range of q-value thresholds spanning from 0.05 to 0.45 in increments of 0.1 to identify whether there were significantly amplified or deleted regions within a chromosome. Focal amplification or deletion for all hg19 samples was determined by setting the broad length cutoff to 0.5 and the confidence level to 0.9, with all other parameters restricted to their default values. Differentially expressed genes located in copy number altered regions were called CNA-driven genes. We obtained somatic mutation genes by removing silent mutations from TCGA. Significantly mutated genes with differential expressions were termed mutation-driven genes. These DNA methylation-driven genes, CNA-driven genes and mutation-driven genes were termed genetic alteration-driven gene in BRCA.

### Identifying enriched pathways and functions of genetic alteration-driven genes

To identify enriched Kyoto Encyclopedia of Genes and Genomes (KEGG) pathways of genetic alteration-driven genes, we performed analyses using the Database for Annotation, Visualization and Integrated Discovery (DAVID)^78^, which conducts a statistical test based on Fisher's exact test to measure gene-enrichment in annotation terms. KEGG pathways were considered to be significant if they had a reported FDR < 0.05 and enrichment scores higher than 1. We performed analyses using KEGG Orthology Based Annotation System (KOBAS 3.0)^79^, a functional enrichment analysis tool, to identify enriched Gene Ontology (GO) terms for genetic alteration-driven genes. A hypergeometric test was used to determine the significance of the results obtained and p-values were corrected using FDR, with an FDR < 0.05 considered significant.

### Identifying functional regulations between genetic alteration-driven genes and drug target genes in breast cancer

The mutual predictability method was used to characterize the functional correlation between genetic alteration-driven genes and DRGs in BRCA. A weighted functional linkage network in which a linked gene pair showed high probability sharing the same biological process was obtained from the visANT database (Integrative Visual Analysis Tool for Biological Networks and Pathways; http://www.visantnet.org/visantnet.html)^80^. We used the mutual predictability to measure the extent to which genetic alteration-driven genes could be used to identify BRCA DRGs, and vice versa. For genetic alteration-driven genes, we identified and ranked their direct neighbors using the weight of edges in the functional linkage network. A receiver operating characteristic (ROC) plot showed the sensitivity and specificity variation by using different weight cutoffs on the ranked list. The true (or false) positive rates represent BRCA DRGs (or non-DRGs) above a particular weight cutoff, respectively. The true (or false) negative rates represent non-DRGs (or BRCA DRGs) below a particular weight cutoff, respectively. The area under the ROC curve (AUC) values were used to evaluate the predictive performance of genetic alteration-driven genes for BRCA DRGs. Similarly, we also calculated AUC values to evaluate the predictive performance of BRCA DRGs as for genetic alteration-driven genes. Finally, the geometric mean of mutual predicted AUC values was used as a mutual predictability score to characterize the functional correlation between genetic alteration-driven gene and DRG in BRCA. The top 100 functional correlations between genetic alteration-driven genes and DRGs using mutual predictability scores were selected as candidate functional gene pairs in BRCA for subsequent analysis.

### Identifying functional regulations between genetic alteration-driven genes and drug response genes acting as prognostic biomarkers in breast cancer

First, univariate Cox regression analyses were conducted for genetic alteration-driven gene and DRG with their expression values as variables. The effect of the genes with *P*-value < 0.1 at the univariate analysis on the overall outcome was tested in multivariate Cox regression analysis. For each functional regulation between a genetic alteration-driven gene and a DRG, a multivariate Cox regression was performed to calculate the contribution of each gene to survival prediction. The risk score of each functional regulation was established based on a linear combination of the expression levels and the multivariable Cox regression coefficient as a weight. Subsequently, multivariable Cox proportional hazard regression analysis was performed to evaluate whether functional regulations could be independent of other clinicopathological variables including age (young ≤ 55; old > 55), ER status, PR status and pathologic stage (early stage I/II; late stage III/IVV). According to the median risk score of selected functional regulations, the patients with BRCA were classified into a high-risk group and a low-risk group. Kaplan–Meier survival curves were then generated, and a log-rank test was performed to reveal alterations in survival time between patients using the R package survival and survminer. Functional regulations between genetic alteration-driven genes and DRGs with FDR < 0.05 were identified as prognostic biomarkers of BRCA. Time-dependent ROC curve analyses were made to assess the predictive capacity of each gene pair by using the R package timeROC.

### Ethical compliance

All methods were performed in accordance with the relevant guidelines.

## Supplementary Information

Supplementary Figures.

## Data Availability

The datasets generated and/or analyzed during the current study are available in the TCGA repository (https://portal.gdc.cancer.gov/).

## References

[1] Aben N, Vis DJ, Michaut M, Wessels LF (2016). TANDEM: A two-stage approach to maximize interpretability of drug response models based on multiple molecular data types. Bioinformatics.

[2] Wang L (2019). Systematic identification of lincRNA-based prognostic biomarkers by integrating lincRNA expression and copy number variation in lung adenocarcinoma. Int. J. Cancer.

[3] Zhao H (2020). LncTarD: A manually-curated database of experimentally-supported functional lncRNA-target regulations in human diseases. Nucleic Acids Res..

[4] Duijf PHG (2019). Mechanisms of genomic instability in breast cancer. Trends Mol. Med..

[5] Kalimutho M (2019). Patterns of genomic instability in breast cancer. Trends Pharmacol. Sci..

[6] Petrucelli N, Daly MB, Pal T, Adam MP (1993). Gene Reviews((R)).

[7] Friedenson B (2007). The BRCA1/2 pathway prevents hematologic cancers in addition to breast and ovarian cancers. BMC Cancer.

[8] O'Donovan PJ, Livingston DM (2010). BRCA1 and BRCA2: Breast/ovarian cancer susceptibility gene products and participants in DNA double-strand break repair. Carcinogenesis.

[9] Kim KH, Roberts CW (2016). Targeting EZH2 in cancer. Nat. Med..

[10] Yoo KH, Hennighausen L (2012). EZH2 methyltransferase and H3K27 methylation in breast cancer. Int. J. Biol. Sci..

[11] Wu Z (2020). Copy number amplification of DNA damage repair pathways potentiates therapeutic resistance in cancer. Theranostics.

[12] Weiss J, Peifer M, Herling CD, Frenzel LP, Hallek M (2019). Acquisition of the recurrent Gly101Val mutation in BCL2 confers resistance to venetoclax in patients with progressive chronic lymphocytic leukemia (Comment to Tausch et al.). Haematologica.

[13] Panagopoulou M (2019). Circulating cell-free DNA in breast cancer: Size profiling, levels, and methylation patterns lead to prognostic and predictive classifiers. Oncogene.

[14] Constancio V, Nunes SP, Henrique R, Jeronimo C (2020). DNA methylation-based testing in liquid biopsies as detection and prognostic biomarkers for the four major cancer types. Cells.

[15] Gyorffy B (2016). Aberrant DNA methylation impacts gene expression and prognosis in breast cancer subtypes. Int. J. Cancer.

[16] Veeck J (2006). Aberrant methylation of the Wnt antagonist SFRP1 in breast cancer is associated with unfavourable prognosis. Oncogene.

[17] Ugolini F (2001). WNT pathway and mammary carcinogenesis: Loss of expression of candidate tumor suppressor gene SFRP1 in most invasive carcinomas except of the medullary type. Oncogene.

[18] Suzuki H (2008). Frequent epigenetic inactivation of Wnt antagonist genes in breast cancer. Br. J. Cancer.

[19] Kong L, Yang W, Chen L, Qian L (2021). The DNA methylation-regulated MCTP1 activates the drug-resistance of esophageal cancer cells. Aging (Albany NY).

[20] Zhao H (2021). Comprehensive landscape of epigenetic-dysregulated lncRNAs reveals a profound role of enhancers in carcinogenesis in BC subtypes. Mol. Ther. Nucleic Acids.

[21] Chang Y (2018). Cancer drug response profile scan (CDRscan): A deep learning model that predicts drug effectiveness from cancer genomic signature. Sci. Rep..

[22] Volckmar AL (2018). A field guide for cancer diagnostics using cell-free DNA: From principles to practice and clinical applications. Genes Chromosomes Cancer.

[23] Ahn S, Woo JW, Lee K, Park SY (2020). HER2 status in breast cancer: Changes in guidelines and complicating factors for interpretation. J. Pathol. Transl. Med..

[24] Cortesi L, Rugo HS, Jackisch C (2021). An overview of PARP inhibitors for the treatment of breast cancer. Target Oncol..

[25] Huang CC (2022). Prevalence of tumor genomic alterations in homologous recombination repair genes among Taiwanese breast cancers. Ann. Surg. Oncol..

[26] Bitler BG (2015). Synthetic lethality by targeting EZH2 methyltransferase activity in ARID1A-mutated cancers. Nat. Med..

[27] Jadhav RR (2015). Genome-wide DNA methylation analysis reveals estrogen-mediated epigenetic repression of metallothionein-1 gene cluster in breast cancer. Clin. Epigenet..

[28] Bortolotto LF (2017). Cytotoxicity of trans-chalcone and licochalcone A against breast cancer cells is due to apoptosis induction and cell cycle arrest. Biomed. Pharmacother..

[29] Wang-Johanning F (2012). Immunotherapeutic potential of anti-human endogenous retrovirus-K envelope protein antibodies in targeting breast tumors. J. Natl. Cancer Inst..

[30] Jia Y, Wang Y, Zhang C, Chen MY (2020). Upregulated CBX8 promotes cancer metastasis via the WNK2/MMP2 pathway. Mol. Ther. Oncolytics.

[31] Zhou Y (2020). A novel bispecific antibody targeting CD3 and prolactin receptor (PRLR) against PRLR-expression breast cancer. J. Exp. Clin. Cancer Res..

[32] Yamauchi T (2003). Cloning of adiponectin receptors that mediate antidiabetic metabolic effects. Nature.

[33] Chung SJ (2017). ADIPOQ/adiponectin induces cytotoxic autophagy in breast cancer cells through STK11/LKB1-mediated activation of the AMPK-ULK1 axis. Autophagy.

[34] Matsuo T (2014). Involvement of B3GALNT2 overexpression in the cell growth of breast cancer. Int J Oncol.

[35] Tsai HT (2020). Multi-gene signature of microcalcification and risk prediction among Taiwanese breast cancer. Sci. Rep..

[36] Jansson AK, Gunnarsson C, Cohen M, Sivik T, Stal O (2006). 17beta-hydroxysteroid dehydrogenase 14 affects estradiol levels in breast cancer cells and is a prognostic marker in estrogen receptor-positive breast cancer. Cancer Res..

[37] Christie EL (2019). Multiple ABCB1 transcriptional fusions in drug resistant high-grade serous ovarian and breast cancer. Nat. Commun..

[38] Lawlor D (2014). PARP inhibitors as P-glyoprotein substrates. J. Pharm. Sci..

[39] Long X (2019). Long non-coding RNA GAS5 inhibits DDP-resistance and tumor progression of epithelial ovarian cancer via GAS5-E2F4-PARP1-MAPK axis. J. Exp. Clin. Cancer Res..

[40] Zuo H (2020). Differential regulation of breast cancer bone metastasis by PARP1 and PARP2. Nat. Commun..

[41] Thomas R, Al-Rashed F, Akhter N, Al-Mulla F, Ahmad R (2019). ACSL1 regulates TNFalpha-induced GM-CSF production by breast cancer MDA-MB-231 cells. Biomolecules.

[42] Tsumagari K (2015). Simultaneous suppression of the MAP kinase and NF-kappaB pathways provides a robust therapeutic potential for thyroid cancer. Cancer Lett..

[43] Yu Q (2016). Resokaempferol-mediated anti-inflammatory effects on activated macrophages via the inhibition of JAK2/STAT3, NF-kappaB and JNK/p38 MAPK signaling pathways. Int. Immunopharmacol..

[44] Reddy D, Kumavath R, Tan TZ, Ampasala DR, Kumar AP (2020). Peruvoside targets apoptosis and autophagy through MAPK Wnt/beta-catenin and PI3K/AKT/mTOR signaling pathways in human cancers. Life Sci..

[45] Fekete JT, Gyorffy B (2019). ROCplot.org: Validating predictive biomarkers of chemotherapy/hormonal therapy/anti-HER2 therapy using transcriptomic data of 3,104 breast cancer patients. Int. J. Cancer.

[46] Han Y (2019). Metformin reverses PARP inhibitors-induced epithelial-mesenchymal transition and PD-L1 upregulation in triple-negative breast cancer. Am. J. Cancer Res..

[47] Peng CL (2021). Metformin relieves H/R-induced cardiomyocyte injury through miR-19a/ACSL axis - possible therapeutic target for myocardial I/R injury. Toxicol. Appl. Pharmacol..

[48] Mai Q (2020). Steroid 5 alpha-reductase 3 (SRD5A3) promotes tumor growth and predicts poor survival of human hepatocellular carcinoma (HCC). Aging.

[49] Li J (2011). Androgen regulation of 5alpha-reductase isoenzymes in prostate cancer: Implications for prostate cancer prevention. PLoS ONE.

[50] Juhasz S (2020). The chromatin remodeler ALC1 underlies resistance to PARP inhibitor treatment. Sci. Adv..

[51] Colic M (2019). Identifying chemogenetic interactions from CRISPR screens with drugZ. Genome Med..

[52] Sakalar C (2016). The combination of thymoquinone and paclitaxel shows anti-tumor activity through the interplay with apoptosis network in triple-negative breast cancer. Tumour Biol..

[53] Xu B (2014). Association of phosphatase and tensin homolog low and phosphatidylinositol 3-kinase catalytic subunit alpha gene mutations on outcome in human epidermal growth factor receptor 2-positive metastatic breast cancer patients treated with first-line lapatinib plus paclitaxel or paclitaxel alone. Breast Cancer Res..

[54] Kavarthapu R, Anbazhagan R, Dufau ML (2021). Crosstalk between PRLR and EGFR/HER2 signaling pathways in breast cancer. Cancers.

[55] Bogorad RL (2008). Identification of a gain-of-function mutation of the prolactin receptor in women with benign breast tumors. Proc. Natl. Acad. Sci. USA.

[56] Goffin V (2017). Prolactin receptor targeting in breast and prostate cancers: New insights into an old challenge. Pharmacol. Ther..

[57] Tan D (2013). Histone trimethylation of the p53 gene by expression of a constitutively active prolactin receptor in prostate cancer cells. Chin. J. Physiol..

[58] Dandawate P (2020). Diphenylbutylpiperidine antipsychotic drugs inhibit prolactin receptor signaling to reduce growth of pancreatic ductal adenocarcinoma in mice. Gastroenterology.

[59] Trott JF (2012). Triennial Lactation Symposium: Prolactin: The multifaceted potentiator of mammary growth and function. J. Anim. Sci..

[60] Nouhi Z (2006). Defining the role of prolactin as an invasion suppressor hormone in breast cancer cells. Cancer Res..

[61] Nitze LM (2013). Reevaluation of the proposed autocrine proliferative function of prolactin in breast cancer. Breast Cancer Res. Treat..

[62] Shams A (2021). Prolactin receptor-driven combined luminal and epithelial differentiation in breast cancer restricts plasticity, stemness, tumorigenesis and metastasis. Oncogenesis.

[63] Lopez-Ozuna VM, Hachim IY, Hachim MY, Lebrun JJ, Ali S (2019). Prolactin modulates TNBC aggressive phenotype limiting tumorigenesis. Endocr. Relat. Cancer.

[64] Guruprasad P, Lee YG, Kim KH, Ruella M (2021). The current landscape of single-cell transcriptomics for cancer immunotherapy. J. Exp. Med..

[65] Nirschl CJ (2017). IFNgamma-dependent tissue-immune homeostasis is co-opted in the tumor microenvironment. Cell.

[66] Qiu Z, Li Y, Zeng B, Guan X, Li H (2018). Downregulated CDKN1C/p57(kip2) drives tumorigenesis and associates with poor overall survival in breast cancer. Biochem. Biophys. Res. Commun..

[67] Lai J (2021). CDKN1C as a prognostic biomarker correlated with immune infiltrates and therapeutic responses in breast cancer patients. J. Cell Mol. Med..

[68] Tot T, Gere M, Hofmeyer S, Bauer A, Pellas U (2021). The clinical value of detecting microcalcifications on a mammogram. Semin. Cancer Biol..

[69] Karamouzis MV (2002). Non-palpable breast carcinomas: Correlation of mammographically detected malignant-appearing microcalcifications and molecular prognostic factors. Int. J. Cancer.

[70] Rojo F (2012). Nuclear PARP-1 protein overexpression is associated with poor overall survival in early breast cancer. Ann. Oncol..

[71] Liao Y, Liao Y, Li J, Xiong J, Fan Y (2020). Polymorphisms in PARP1 predict disease-free survival of triple-negative breast cancer patients treated with anthracycline/taxane based adjuvant chemotherapy. Sci. Rep..

[72] Sobczak M, Pitt AR, Spickett CM, Robaszkiewicz A (2019). PARP1 co-regulates EP300-BRG1-dependent transcription of genes involved in breast cancer cell proliferation and DNA repair. Cancers.

[73] Kim DS (2019). Activation of PARP-1 by snoRNAs controls ribosome biogenesis and cell growth via the RNA helicase DDX21. Mol. Cell.

[74] Michmerhuizen AR (2019). PARP1 inhibition radiosensitizes models of inflammatory breast cancer to ionizing radiation. Mol. Cancer Ther..

[75] Qi L (2019). A four-mRNA model to improve the prediction of breast cancer prognosis. Gene.

[76] Zhang YP (2021). Over-expression of SRD5A3 and its prognostic significance in breast cancer. World J. Surg. Oncol..

[77] Mermel CH (2011). GISTIC2.0 facilitates sensitive and confident localization of the targets of focal somatic copy-number alteration in human cancers. Genome Biol..

[78] da Huang W, Sherman BT, Lempicki RA (2009). Systematic and integrative analysis of large gene lists using DAVID bioinformatics resources. Nat. Protoc..

[79] Xie C (2011). KOBAS 2.0: A web server for annotation and identification of enriched pathways and diseases. Nucleic Acids Res..

[80] Linghu B, Snitkin ES, Hu Z, Xia Y, Delisi C (2009). Genome-wide prioritization of disease genes and identification of disease-disease associations from an integrated human functional linkage network. Genome Biol..

[81] Kanehisa M, Furumichi M, Sato Y, Ishiguro-Watanabe M, Tanabe M (2021). KEGG: Integrating viruses and cellular organisms. Nucleic Acids Res..

