# Supplementary material for: Functional regulations between genetic alteration-driven genes and drug target genes acting as prognostic biomarkers in breast cancer
Source: Sci Rep. 2022 Jun 23;12:10641. doi: 10.1038/s41598-022-13835-5 (PMC9226112; doi:10.1038/s41598-022-13835-5)
Supplement: Supplementary file 1 — Supplementary Figures. [file 41598_2022_13835_MOESM1_ESM.pdf]

Functional regulations between genetic alteration-driven genes and drug target genes acting as prognostic biomarkers in breast cancer

Li Wang [1,$,*], Lei Yu[1,$], Jian Shi[2, $], Feng Li[1, $], Caiyu Zhang[1], Haotian Xu[1], Xiangzhe Yin[1], Lixia Wang[1], Shihua Lin[1], Litvinova Anastasiia[1], Yanyan Ping[1,*], Shangwei Ning[1,*], Hongying Zhao[1,*]

[1]College of Bioinformatics Science and Technology, Harbin Medical University, Harbin 150081, China.

[2]Precision Medicine Institute, The First Affiliated Hospital, Sun Yat-sen University, Guangzhou, China.

[$]These authors contributed equally to this work.

***Corresponding Authors:**

Hongying Zhao, College of Bioinformatics Science and Technology, Harbin Medical University, Harbin 150081, China. Tel.: 86-451-86615922, E-mail: zhaohongying@hrbmu.edu.cn;

Shangwei Ning, College of Bioinformatics Science and Technology, Harbin Medical University, Harbin 150081, China. Tel.: 86-451-86615922, E-mail: ningsw@ems.hrbmu.edu.cn;

Yanyan Ping, College of Bioinformatics Science and Technology, Harbin Medical University, Harbin 150081, China. Tel.: 86-451-86615922, E-mail: pingyanyan@hrbmu.edu.cn;

Li Wang, College of Bioinformatics Science and Technology, Harbin Medical University, Harbin 150081, China. Tel.: 86-451-86615922, wangli@hrbmu.edu.cn.

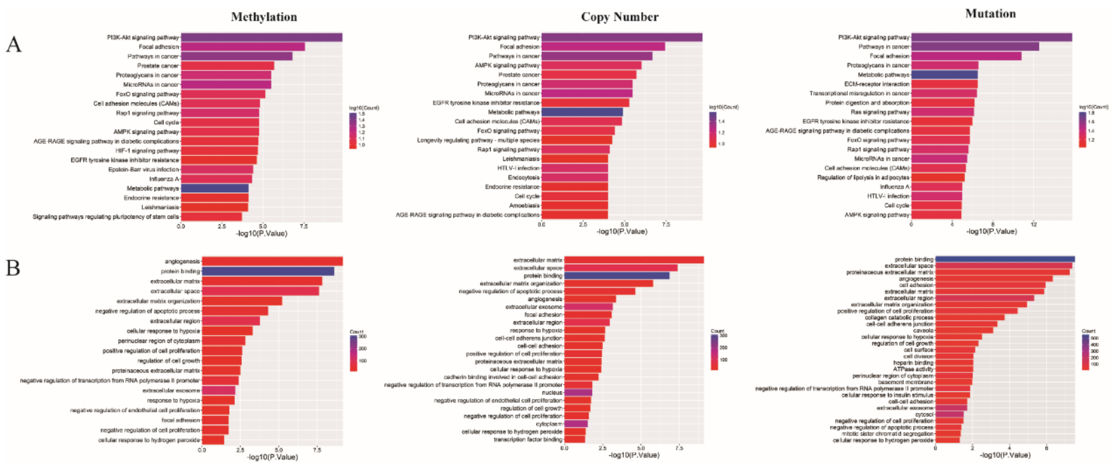

Figure S1. Identification of biological pathways and functions in breast cancer. (A) Methylation, copy number, and mutation with significantly enriched pathways. (B) Methylation, copy number, and mutation with significantly enrichment functions.

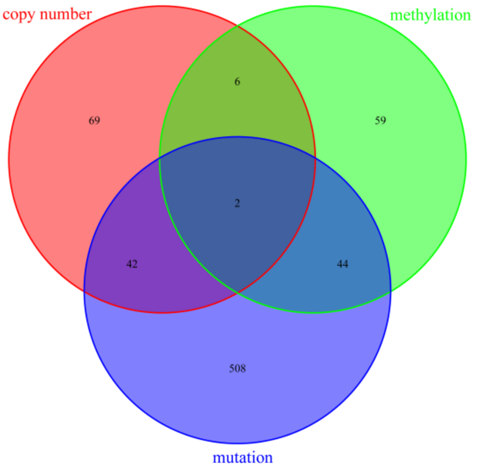

Figure S2. Intersection diagram of genes which methylation alteration-driven, CNV alteration-driven and Mutation alteration-driven.

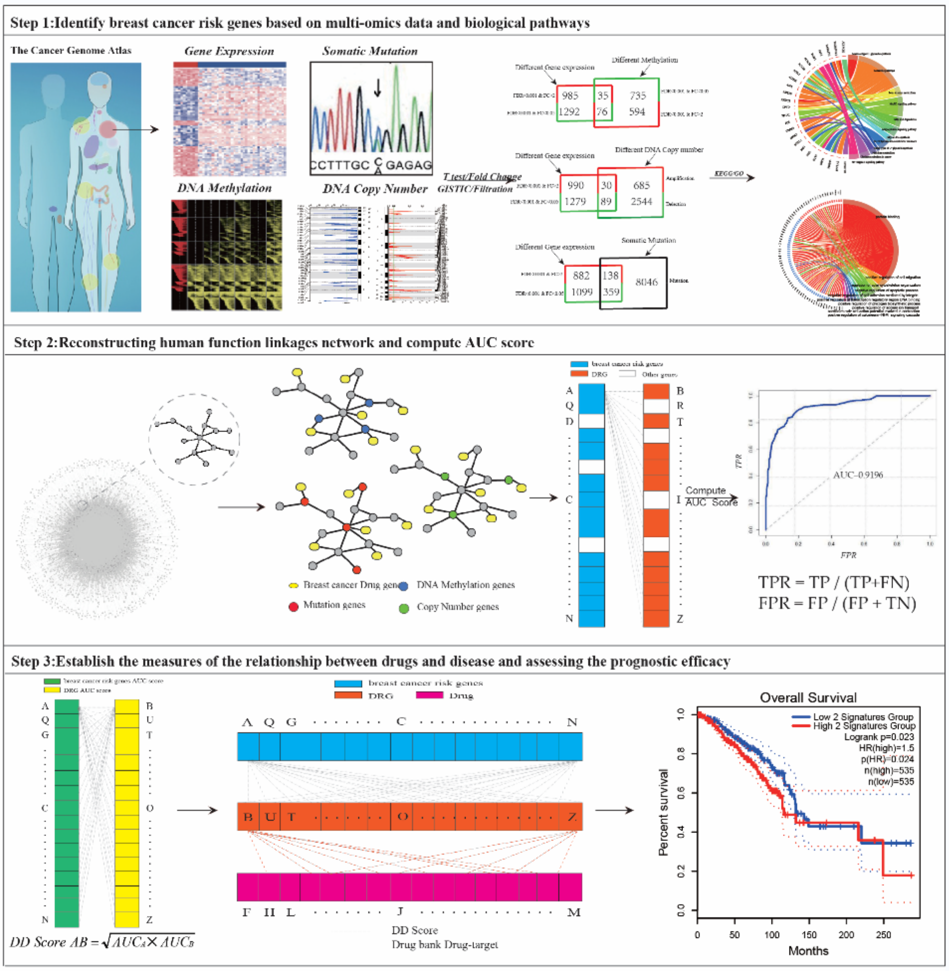

Figure S3. Flowchart of the strategy to identify functional regulations between genetic alteration-driven genes and drug target genes. Mapping the methylation, copy number, and mutation risk genes to the human functional linkage network. mutual predictability method was used to identified functional correlation between genetic alteration-driven genes and drug response genes in breast cancer. The potentially prognostic value of functional regulations between genetic alteration-driven genes and drug target genes were calculated.

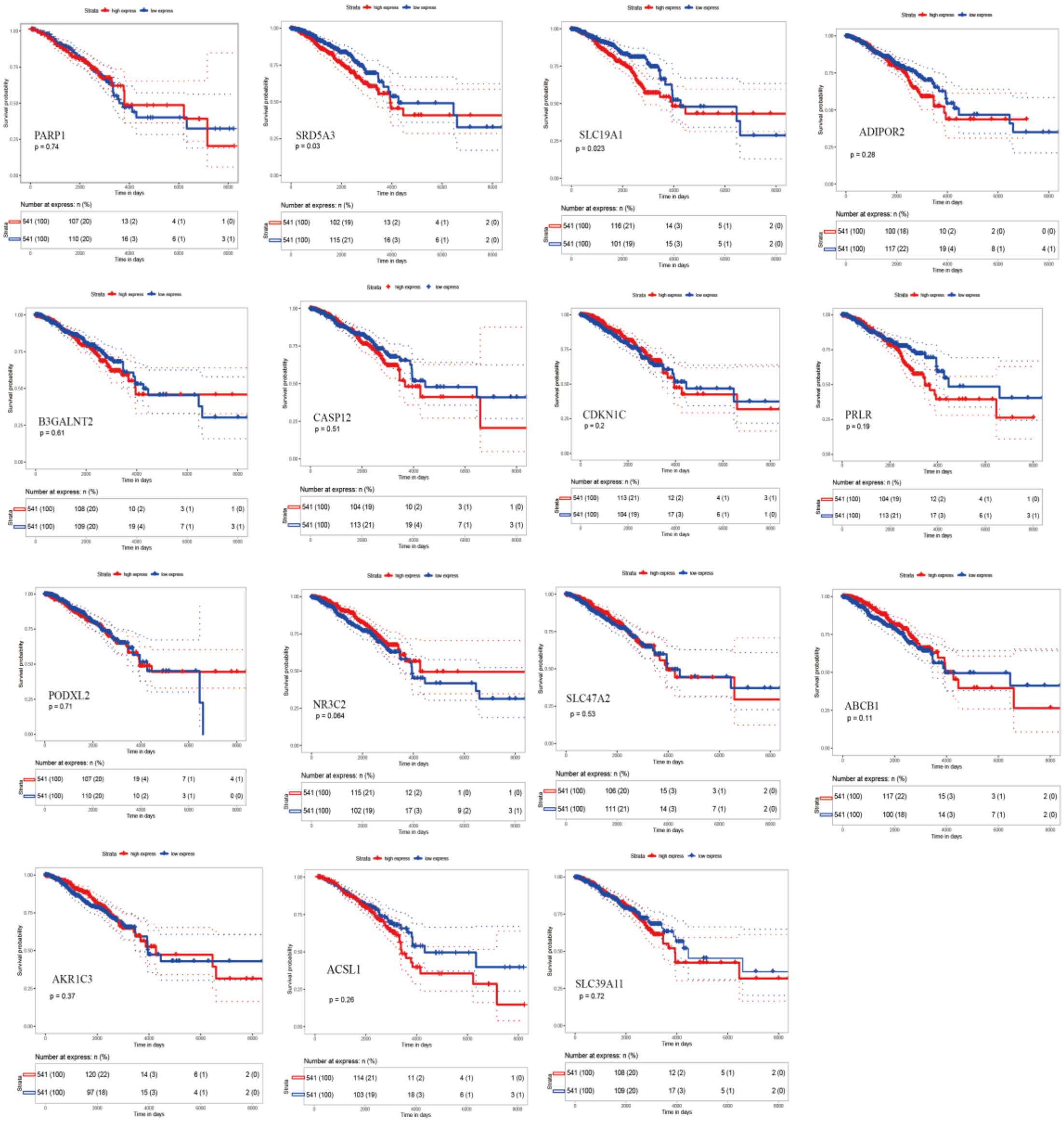

Figure S4. Survival analysis of individual genes of genetic alteration-driven gene pairs in the TCGA cohort. Comparison of the survival rates of two groups with respect to individual genes, which are involved in prognostic genetic alteration-driven gene pairs.
